# Supplementary material for: Use of Early Donated COVID-19 Convalescent Plasma Is Optimal to Preserve the Integrity of Lymphatic Endothelial Cells
Source: Pharmaceuticals (Basel). 2022 Mar 17;15(3):365. doi: 10.3390/ph15030365 (PMC8948637; doi:10.3390/ph15030365)
Supplement: Supplementary file 1 [file pharmaceuticals-15-00365-s001.zip › pharmaceuticals-1621500-supplementary.pdf]

Use of Early Donated COVID-19 Convalescent Plasma Is Optimal to Preserve the Integrity of Lymphatic Endothelial Cells

Supplementary material

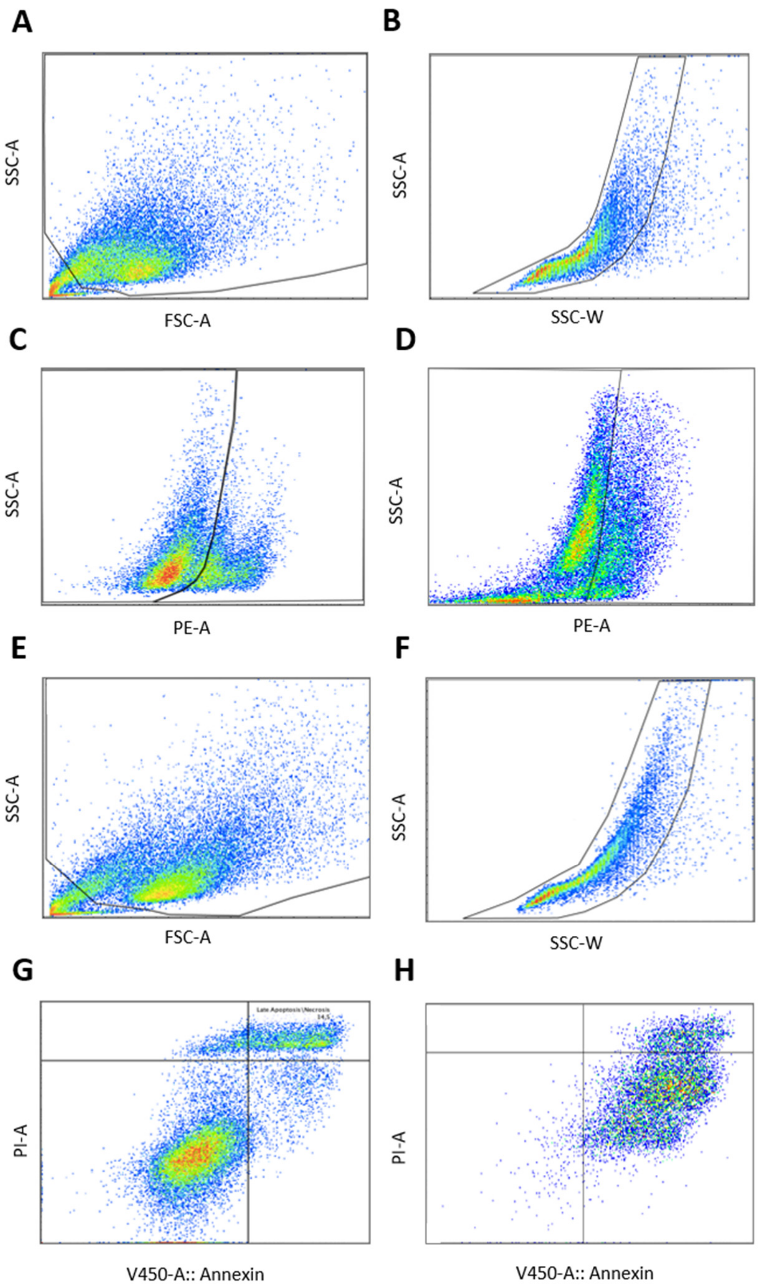

Figure S1. Gating strategy in flow cytometry for reactive oxygen species production and cellular viability. **(A – D).** MitoSOX^{TM} Red gating strategy through viable cells (side scatter-area) (**A**), single cells (side scatter-area) (**B**) MitoSOX^{TM} Red negative and positive populations following incubation with CCP (side scatter-area) (**C**) and MitoSOX^{TM} Red negative and positive populations following incubation with 1mM H_2O_2 (side scatter-area) (**D**). **(E – H)** Cellular viability gating strategy using Annexin V and PI through viable cells (side scatter-area) (**E**), single cells (side scatter-area) (**F**) early apoptosis population (Annexin V positive and negative for PI) and necrosis (Annexin V positive and PI positive) following incubation with CCP (PI - area) (**G**) and MitoSOX^{TM} Red negative and positive

populations following incubation with 1 μM Staurosporine (side scatter-area) (**H**). SSC-A, side scatter-area; FSC-A, forward scatter-area; PI, propidium iodide; CCP, COVID-19 convalescent plasma.

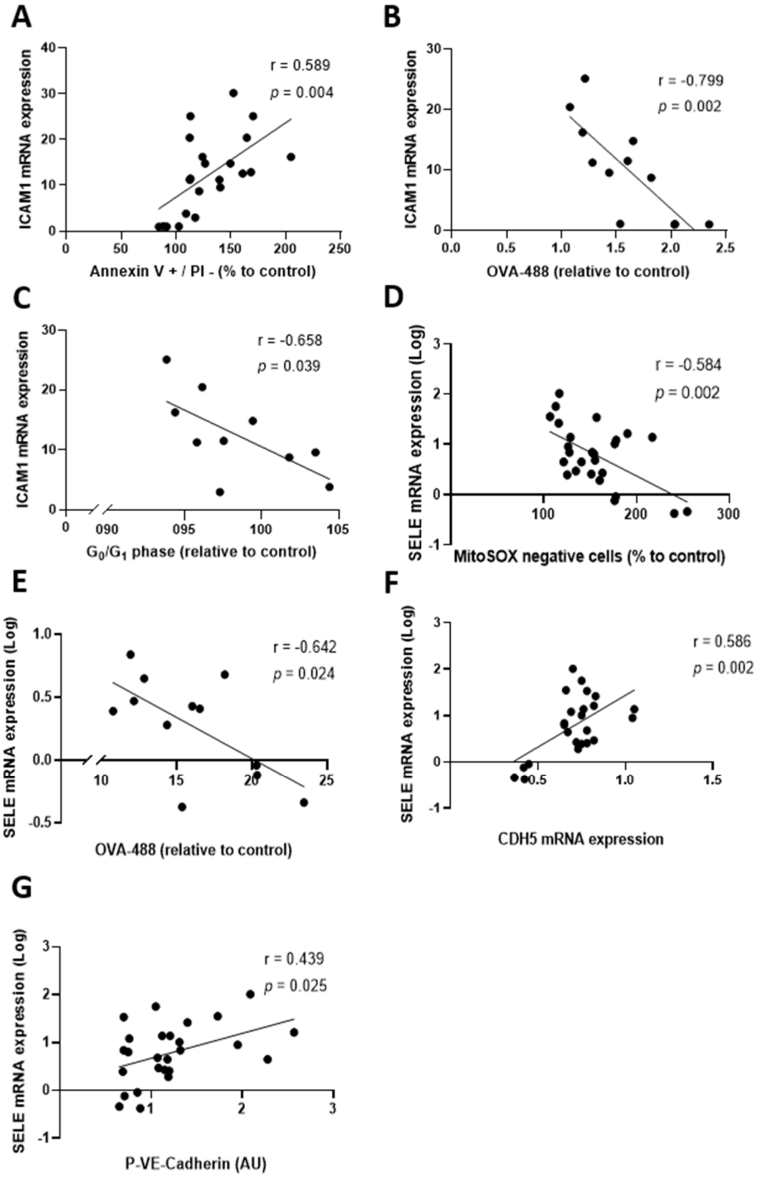

Figure S2. Endothelial activation markers are predictors of preserved endothelial integrity. (**A** – **C**) Treated aHDLEC (convalescent plasma for 4h and cytokines for 20h) were harvested and mRNA expression of activation marker *ICAM1* was assessed by RT-qPCR and correlated with cells in early apoptosis (positive for Annexin V and negative for propidium iodide (PI) (**A**), concentration of ovalbumin-488 following the permeability transwell test (**B**) and cells in the G_0/G_1 phase of the cell cycle, determined by DNA fragmentation using PI (**C**). (**D** – **G**). Treated aHDLEC were harvested and mRNA expression of activation marker E-selectin (*SELE*) was determined by RT-qPCR and correlated with cells not labeled by the MitoSOX^{TM} Red probe (**D**), concentration of ovalbumin-488 (**E**), VE-Cadherin mRNA expression (**F**) and phosphorylated VE-Cadherin expression measured by immunoblot and normalized to the expression of non-phosporylated VE-Cadherin (**G**). Significance was determined by a Pearson correlation

and $p < 0,05$ was considered significant. *ICAM1*, gene coding for intercellular adhesion molecule 1; *SELE*, gene coding for E-selectin; PI, propidium iodide; *CDH5*, gene coding for VE-Cadherin; OVA, ovalbumin; P-VE-Cadherin, phosphorylated VE-Cadherin; aHDLEC, adult human dermal lymphatic endothelial cells.

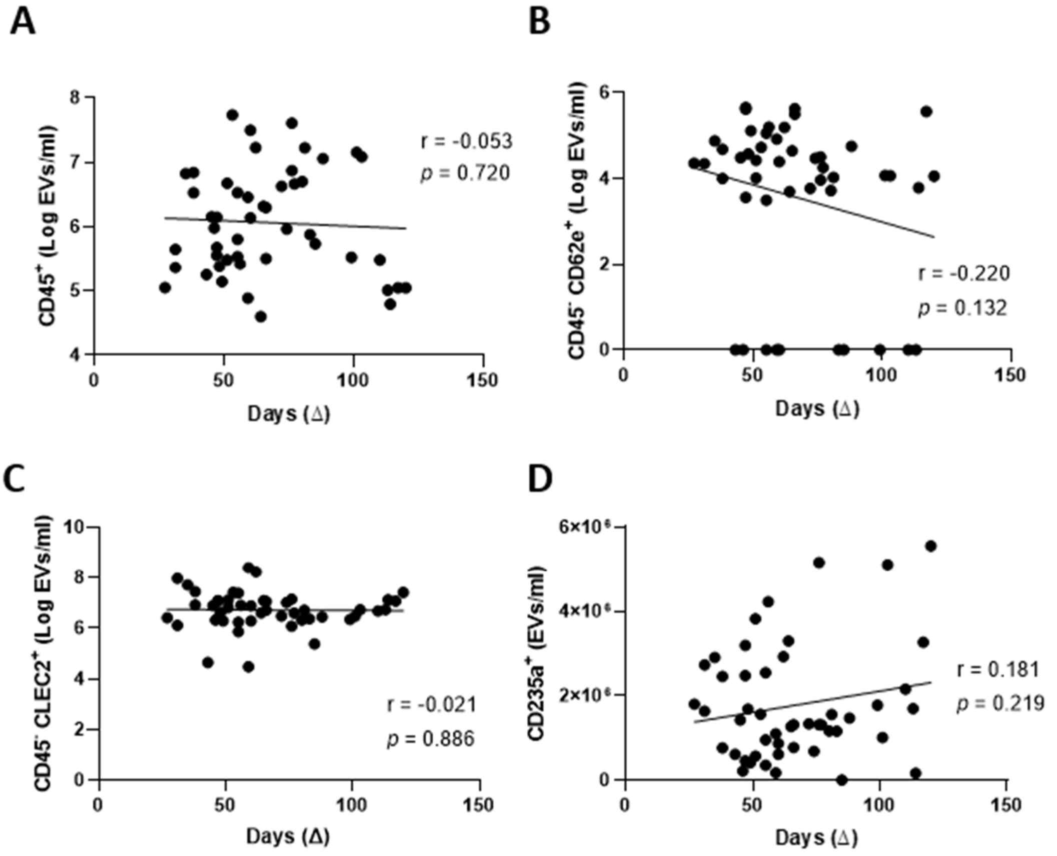

Figure S3. No significant correlations between extracellular vesicles and time of donation of COVID-19 convalescent plasma. (**A – D**) Correlation between the duration between the onset of symptoms and the donation and the concentration of CD45^{+} EVs (**A**), CD45^{+} CD62e^{+} EVs (**B**), CD45^{-} CLEC2^{+} EVs (**C**) and CD235a^{+} EVs in CCP (**D**). Significance was determined by a Pearson correlation after a logarithmic transformation of CD45^{+} EVs, CD45^{-} CD62e^{+} EVs and CD45^{-} CLEC2^{+} EVs concentration to reach a normal distribution. $p < 0,05$ was considered significant. Δ, time of donation since onset of symptoms; EVs, extracellular vesicles; CLEC2, C-type lectin-like type II; CCP, COVID-19 convalescent plasma.

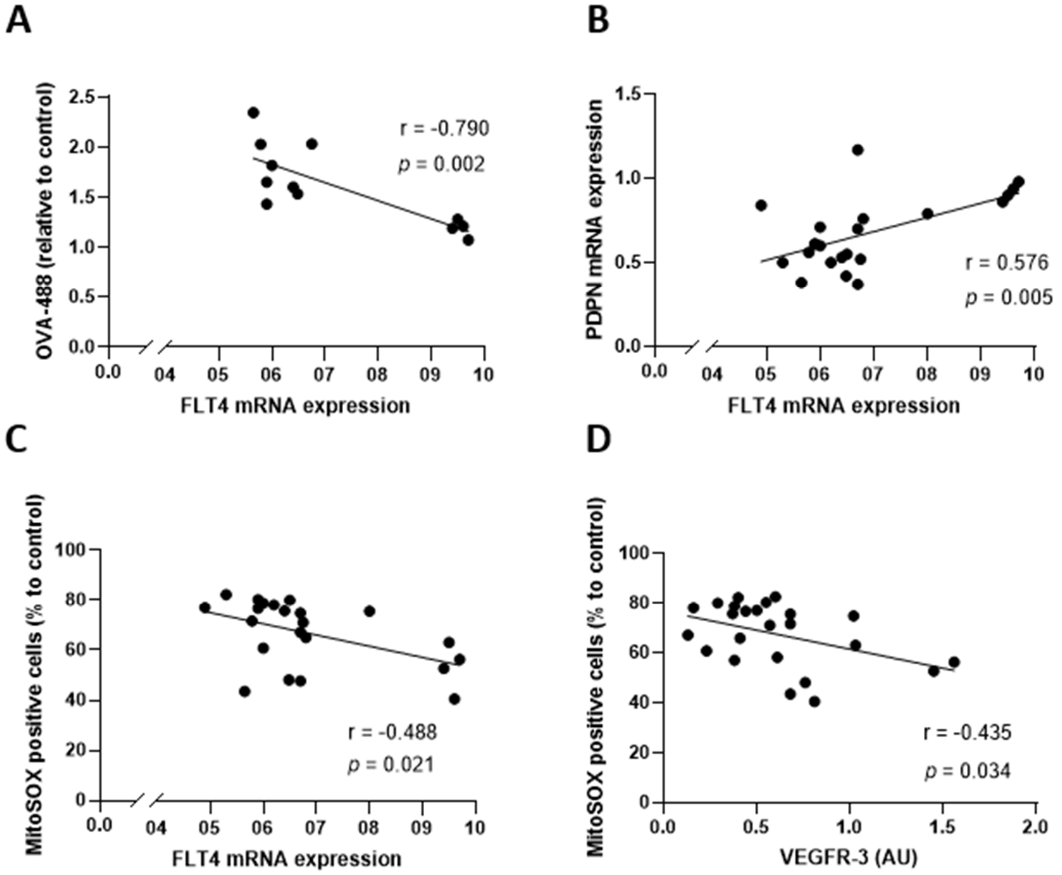

Figure S4. VEGFR-3 mRNA expression correlates to a preserved endothelial integrity. (**A**) Treated cells (convalescent plasma for 4h and cytokines for 20h) were harvested and mRNA expression of *FLT4* was assessed by RT-qPCR and correlated with the concentration of ovalbumin-488 measured following the permeability transwell assay. (**B**) The mRNA expression of FLT-4 was correlated with the mRNA expression of podoplanin in treated cells (**C**) The mRNA expression of FLT4 was correlated with cells labeled by the MitoSOX^{TM} Red probe (relative to cells treated with control plasma) (**D**) Proteins from treated cells were extracted and the expression of VEGFR-3 was assessed by immunoblot and correlated with cells labeled by the MitoSOX^{TM} Red probe (relative to cells treated with control plasma). Significance was determined by a Pearson correlation and $p < 0,05$ was considered significant. OVA, ovalbumin; FLT4, gene coding for VEGFR-3; VEGFR-3, vascular endothelial growth factor receptor 3; PDPN; gene coding for podoplanin.

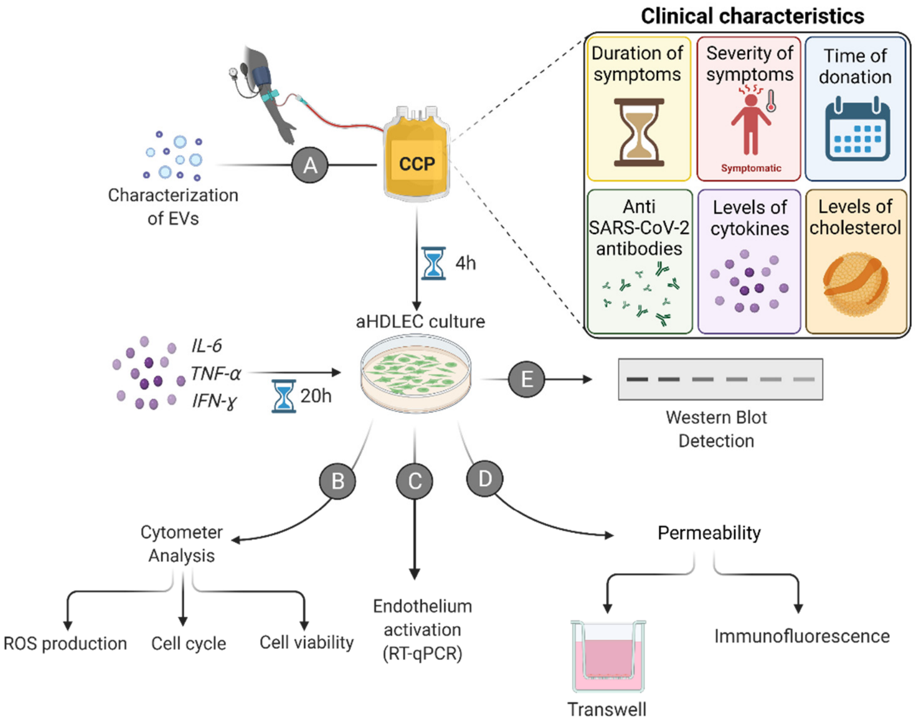

Figure S5. Experimental design. **(A – E)** Clinical characteristic of COVID-19 convalescent plasma donors were assessed. EVs within CCP were characterized (**A**). Following the incubation of CCP on aHDLEC, cells were treated with a pro-inflammatory cocktail of cytokines (IFNɣ [10 ng/ml], IL-6 [20 ng/ml] and TNFα [20 ng/ml]). Flow cytometry analysis for cell viability, mitochondrial ROS production and cell cycle were performed (**B**). Endothelial activation (**C**), permeability (**D**), and protein expression (**E**) were also analyzed. CCP, COVID-19 convalescent plasma; EVs, extracellular vesicles; aHDLEC, adult human dermal lymphatic endothelial cells; ROS, reactive oxygen species; IFNɣ, interferon gamma; IL-6, interleukin 6; TNFα, tumor necrosis factor alpha. *Created with BioRender.com*

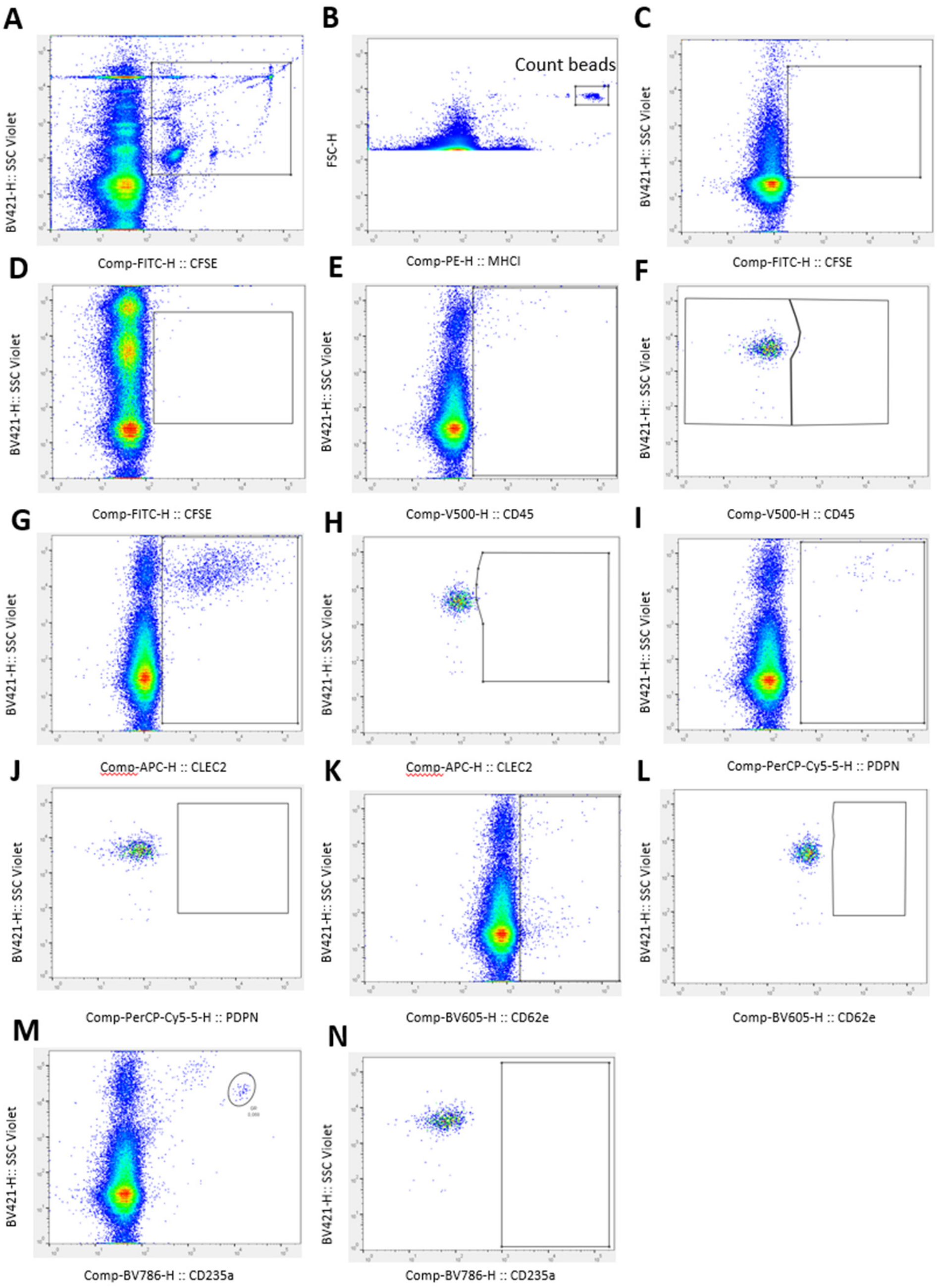

Figure S6. Flow cytometry plots gating for the identification of extracellular vesicles. (**A**) Megamix (180- to 1300-nm silica beads and 110- and 500-nm fluorescent latex Apogee beads) and filter of 405/40 gated with CFSE (side scatter-height). (**B**) Count Beads (3-µm-diameter MP-CB) (forward scatter-height). (**C**) Background sample used to eliminate background noise (side scatter-height). (**D**) Unlabeled sample used as unstained sample for gating strategy (side scatter-height). Single stained samples were used for gating strategy. As controls, detergent Triton X-100 was added to the antibody mix to verify the membrane of EVs. Single stained (**E**) and depletion using Triton X-100 (**F**) of $CD45^+$ EVs. Single stained (**G**) and depletion (**H**) of $CLEC2^+$ EVs. Single stained (**I**) and depletion (**J**) of

podoplanin$^+$ EVs. Single stained (**K**) and depletion (**L**) of CD62e$^+$ EVs. Single stained (**M**) and depletion (**N**) of CD235a$^+$ EVs**.** SSC, side scatter; FSC-H, forward scatter-height; CFSE, carboxyfluorescein succinimidyl ester; MHCI, major histocompatibility complex I; PDPN, podoplanin; CLEC2, C-type lectin-like type II; EVs, extracellular vesicles.

Table S1 List of conjugated antibodies and markers used for flow cytometry

Antibodies/ Proteins	Fluoro.	Excitation (nm)	Emission (nm)	Company	Catalogue	Dilution or Conc.
**CFSE**	FITC	488	530/30	Cedarlane, Burlington, Ontario, Canada,	S8269-10MG	1/5000
**MHC I**	PE	488	575/25	BD Biosciences, Franklin Lakes, NJ, USA	555553	1/200
**CD235a**	BV786	405	780/60	BD Biosciences, Franklin Lakes, NJ, USA	740984	1/500
**CD45**	V500	405	525/50	BD Biosciences, Franklin Lakes, NJ, USA	560777	1/100
**CLEC2**	APC	640	670/30	BioLegend, San Diego, CA, USA	372005	1/50
**CD62e**	BV605	405	610/20	BD Biosciences, Franklin Lakes, NJ, USA	563359	1/75
**Podoplanin**	PerCP-Cy5.5	488	695/40	BioLegend, San Diego, CA, USA	337011	1/100
**Annexin V**	V450	405	450/40	BD Biosciences,	560506	1μL/test

			Franklin Lakes, NJ, USA		
**PI***	488	610/20	Biotium, Fremont, CA, USA	40017	2,5µg/mL
**PI****	488	610/20	Sigma, St. Louis, Missouri, USA	P4170-25MG	50µg/mL
**MitoSOX™ Red**	510	575/25	Thermo Fisher, Waltham, MA, USA	M36008	1,67µM

Fluoro, fluorochrome; Conc, concentration; CFSE, carboxyfluorescein succinimidyl ester; MHC I, major histocompatibility complex I; CLEC2, C-type lectin-like type II; PI*, propidium iodide used for cell viability; PI**, propidium iodide used for cell cycle distribution.

Table S2 List of sequences for PCR primers used throughout the study

Genes	Primers Sequence (5' → 3')
ACTB	**F : GACGACATGGAGAAAATCTG**<br>**R : ATGATCTGGGTCATCTTCTC**
ICAM1	**F : ACCATCTACAGCTTTCCG**<br>**R : TCACACTTCACTGTCACC**
SELE	**F : GAGAATTCACCTACAAGTCC**<br>**R : AGGCTTGAACATTTTACCAC**
FLT4	**F : GAGCAGATAGAGAGCAGGCAT**<br>**R : ACATTCTGGCCAGGTCCTTTAC**
PDPN	**F : AAGATGGTTTGTCAACAGTG**<br>**R : GTACCYYCCCGACATTTTTC**
CDH5	**F : CGCAATAGACAAGGACATAAC**<br>**R : TATCGTGATTATCCGTGAGG**

F, forward; R, reverse; *ACTB*, gene coding for ß-actin; *ICAM1*, gene coding for intercellular adhesion molecule 1; *SELE*, gene coding for E-selectin; *FLT4*, gene coding for vascular endothelial growth factor receptor 3; *PDPN*, gene coding for podoplanin; *CDH5*, gene coding for VE-Cadherin.

Questionnaire

Multicentre Study to Evaluate the Safety and Efficacy of Convalescent Plasma in the Treatment of COVID-19: Convalescent Plasma Donor Component.

**Participant ID number**	________________________________

**Origin of infection**	
Travel abroad	☐
Community transmission	☐
Unknown	☐

**Symptoms**	
Date of onset of symptoms	______/______/______
Date of positive test result	______/______/______
End date of symptoms	______/______/______

**Symptoms: types and severity**	
Overall health condition	☐ Fever<br>☐ Fatigue
Respiratory	☐ Cough<br>☐ Difficulty breathing
Digestive	☐ Nausea<br>☐ Stomach ache<br>☐ Diarrhea
ENT	☐ Loss of smell/taste<br>☐ Nasal congestion
Severity of symptoms	☐ Mild<br>☐ Moderate<br>☐ Strong

Hospitalization	
Were you hospitalized for COVID-19?<br>If you answered Yes :	☐ Yes<br>☐ No
Date of hospitalization	______/______/______
Date of discharge from hospital	______/______/______
**Intensive Care**	
Were you admitted to intensive care?	☐ Yes<br>☐ No
If yes, how many days were you in intensive care?	________________________________
Did you require mechanical ventilation/Were you intubated?	☐ Yes<br>☐ No

Post-test	
Were you tested for the virus after symptoms ceased/disappeared?	☐ Yes<br>☐ No
If yes, what was the result of this test?	☐ Positive<br>☐ Negative

**Completed by:**<br>____	**DATE :**<br>______/______/______<br>**DATE:**<br>______/______/______
